# Is peer support beneficial for individuals with borderline personality disorder? Exploring its potential and challenges – a scoping review

**DOI:** 10.3389/fpsyt.2025.1663685

**Published:** 2025-10-23

**Authors:** Anne-Sophie Dufour, Laurie Nadeau, Lionel Cailhol

**Affiliations:** ^1^ Department of Psychology, University of Montreal, Montreal, QC, Canada; ^2^ Department of Psychology, University of Quebec in Montreal, Montreal, QC, Canada; ^3^ Department of Psychiatry and Addictology, University of Montreal, Montreal, QC, Canada; ^4^ Research Center of the University Institute of Mental Health of Montreal, Montreal, QC, Canada; ^5^ CERVO, Quebec City, QC, Canada

**Keywords:** borderline personality disorder, peer support, peer-led programs, psychosocial support, scoping review

## Abstract

**Background:**

Borderline Personality Disorder (BPD) is associated with profound emotional dysregulation, interpersonal difficulties, and a heightened risk of suicide. Although psychotherapy remains the first-line treatment, systemic limitations such as resource shortages and high dropout rates necessitate exploration of alternative or complementary interventions. In this context, peer support, defined as assistance provided by individuals with lived experience of mental health challenges, has gained interest as a promising complement or alternative to conventional care. Despite its growing use across mental health contexts, its relevance, effectiveness, and limitations remain poorly understood for individuals with BPD.

**Objective:**

This scoping review aims to synthesize the current literature on peer support interventions for individuals with BPD, focusing on their benefits, risks, and implementation challenges.

**Methods:**

Following the Joanna Briggs Institute (JBI) methodology and PRISMA-ScR guidelines, a comprehensive search was conducted across six databases (Medline, PsycINFO, EMBASE, Cochrane Library, CINAHL, and Google Scholar) and grey literature sources. The inclusion criteria were defined using the Population–Concept–Context (PCC) framework. Eleven studies published between 2019 and 2025 were included. Data extraction focused on study design, participant characteristics, intervention content and structure, outcomes, and implementation challenges.

**Results:**

Peer support interventions varied from structured and manualized programs to flexible and community-based formats. Reported benefits included improved emotion regulation, reduced isolation, enhanced empowerment, and increased hope. Peer workers also reported personal growth and a strengthened sense of purpose. However, challenges such as emotional exhaustion, role ambiguity, inadequate supervision, and limited engagement in online settings were frequently noted. Only a few studies included quantitative measures of symptom change, and methodological heterogeneity limited cross-study comparisons.

**Conclusions:**

Peer support interventions show promising psychosocial benefits for individuals with BPD, particularly in domains related to relational connectedness, emotional coping, and subjective recovery. However, evidence remains limited by a lack of standardized models and rigorous evaluation. Future studies should employ mixed-method and controlled designs to better assess clinical outcomes and ensure safe, effective, and sustainable peer-led programs for this population.

## 1 Introduction

Borderline personality disorder (BPD) is characterized by emotional instability, tumultuous interpersonal relationships, marked impulsivity, intense fear of abandonment, and self-destructive behaviors (1). These symptoms can significantly disrupt daily functioning and diminish quality of life (2). Individuals living with BPD often struggle to maintain consistent relationships (3), manage daily responsibilities and regulate their emotions effectively (4, 5). These challenges can lead to considerable difficulties in various aspects of life, including their personal, social, and professional spheres, ultimately reducing their overall sense of fulfillment and well-being (6). Additionally, individuals with BPD are at increased risk for premature death, primarily due to elevated suicide rates and high burden of physical health complications (7, 8). BPD is estimated to affect approximately 1.9% of the general population (9) but accounts for 15–28% of patients in psychiatric settings, including clinics and hospitals, as well as a significant number of individuals seeking psychological support in general healthcare facilities (10).

### 1.1 Challenges in accessing and retaining effective treatment for BPD

Clinical guidelines generally recommend psychotherapy as the first-line treatment for BPD, with Dialectical Behavior Therapy (DBT) often highlighted, despite no consensus regarding the superiority of any psychological intervention (11). However, access to adequate treatment for BPD remains limited in most countries because of significant shortage of qualified professionals, insufficient funding and ongoing stigmatization of this population within the healthcare system (12–14). As a result, many individuals are left without access to the recommended care, as the demand for these services far exceeds the available supply (13). Individuals with BPD frequently report dissatisfaction with the services they receive, citing a significant disparity between their needs and the care provided, while also facing critical stigmatization (15). Although DBT and other psychotherapies are recognized as the most effective treatments, meta-analyses have shown only moderate effect sizes in reducing BPD symptoms with psychotherapy (16). Additionally, a meta-analysis indicates that the dropout rate for outpatient psychotherapies is 28.2% (17), highlighting the challenges of retaining patients in these therapeutic programs.

### 1.2 Alternative and complementary treatment: peer support

Given these limitations, it is crucial to explore complementary approaches that could help bridge the gap in BPD care, improving both accessibility and patient engagement in therapeutic programs. Peer support, defined as the provision of emotional, social, and practical assistance by individuals with lived experience of mental health challenges (18), has gained recognition as a valuable alternative to traditional clinical care for people with mental health conditions (19). By engaging with peers who have faced similar challenges, individuals can experience a sense of validation and support, possibly enhancing their commitment into treatment (19, 20). This peer-based approach not only provides practical coping strategies, but also creates a relational dynamic grounded in shared experience, fostering understanding and empathy that may be harder to cultivate in traditional professional settings (21, 22). Indeed, individuals with BPD often struggle with feelings of isolation (23), a lack of understanding from their social circles (24) and tend to have smaller social networks, increased loneliness and lower-quality relationships (25). Support groups and structured social interactions could play a critical role in reducing isolation and enhancing perceived social support among individuals (26, 27). Studies also suggest that peer support programs can improve outcomes such as self-efficacy, empowerment, and hope, all of which are crucial in the recovery process (19, 22). Furthermore, peer support is typically more accessible and cost-effective than traditional therapies (28), making it an invaluable tool for bridging gaps in mental healthcare accessibility and patient engagement. Peer support emerges as a promising intervention for individuals living with BPD. However, despite its potential, its implementation entails specific risks that must be carefully explored. Individuals with BPD may experience significant challenges related to impulsivity (29) and interpersonal functioning (30), which can complicate peer interactions and disrupt group dynamics. These difficulties underscore the importance of identifying and anticipating potential challenges to ensure that peer support remains both safe and therapeutically beneficial in this population.

This study aims to evaluate the potential benefits and challenges of peer support for individuals living with BPD and identify key functional and symptomatic areas that may be impacted by such interventions. Specifically, this research will investigate:

1. the efficacy and risks of peer-support models, exploring various modalities, structures, implementation strategies, and outcomes that have shown promise in supporting individuals with BPD;
2. the structural, logistical, and ethical challenges of implementing peer-support programs for BPD.

By synthesizing existing evidence and addressing these objectives, this study seeks to provide practical insights for clinicians, researchers, and policymakers, supporting the development of structured, evidence-based peer-support interventions to complement traditional therapeutic approaches and better address the specific needs of individuals with BPD.

## 2 Methodology

### 2.1 Protocol

A scoping review methodology was selected to address the objectives of this study, as it enables systematic mapping, exploration and synthesis of existing literature, while also identifying knowledge gaps (31). The review was conducted in accordance with the Joanna Briggs Institute (JBI) guidelines for Scoping Reviews (32) and the Preferred Reporting Items for Systematic Reviews and Meta-analyses extension for scoping review (PRISMA-ScR) checklist (33, 34). The protocol for this review was not registered; however, it is available in French and English upon request with the corresponding author.

### 2.2 Eligibility criteria

The eligibility criteria were defined according to the Population-Concept-Context (PCC) framework (32). Studies not meeting the following criteria were excluded.

#### 2.2.1 Population

Individuals aged 18 or older exhibiting traits of BPD or associated symptoms, identified either through standardized self-report questionnaires or clinical assessment, without requiring a formal diagnosis.

#### 2.2.2 Concept

Studies evaluating specific peer-support programs or interventions with data on intervention outcomes and/or modalities. Interventions or programs had to involve peer support provided by individuals with lived experience of BPD or associated symptoms.

#### 2.2.3 Context

No restrictions were set regarding the context (inpatient, outpatient, community), sex, or geographical location of the study.

#### 2.2.4 Others

Articles in peer-reviewed journals, such as experimental studies (randomized clinical trials, controlled studies), literature reviews, and observational studies (cohort, case-control), were included, as well as grey literature (e.g., conference abstracts, theses) published in English or French.

### 2.3 Search strategy

Prior to conducting this scoping review, a preliminary search was done in Medline, the Cochrane Database of Systematic Reviews, Embase, and Google Scholar to verify the absence of any existing or ongoing systematic or scoping reviews on the topic. In August 2024, a librarian specializing in knowledge synthesis in mental health at the Research Center of the University Institute of Mental Health in Montreal conducted a comprehensive literature search using the Medline, PsycINFO, EMBASE, Cochrane Library, Google Scholar, and CINAHL databases to identify peer-reviewed articles. The search was limited to publications from the past 20 years, from 2004 to 2024, to capture recent advancements, emerging trends, and evolving methodologies related peer support for individuals with BPD. Additionally, a grey literature search was performed using the Google search engine with targeted keywords and Boolean operators. The grey literature search aimed to expand the scope of the analyzed documents and include sources not indexed in traditional scientific databases, such as institutional reports, peer-support initiatives, theses, and government publications. To ensure global coverage and mitigate regional biases in search results, a VPN was used to access content from four continents. The full search strategy for each database and for the grey literature search is detailed in **Supplementary File 1**. In line with best practices for scoping reviews, a final bibliographic alert was conducted just before submission to capture any newly published relevant studies.

### 2.4 Article selection

Search results were imported into Covidence systematic review management software, which automatically identified and removed duplicates (Covidence Systematic Review Software, 2021). Based on predetermined eligibility criteria, two reviewers (A.-S.D. and M.A.) independently screened the titles and abstracts of the remaining records. Prior to the screening process, a pilot test was conducted to ensure a minimum inter-rater agreement of 75%. Discrepancies were resolved by a third reviewer (F.T.). Full texts were then independently assessed by the same two reviewers (A.-S. D.), with discrepancies settled by the third (F.T.)

### 2.5 Data extraction

Data to extract from eligible articles was discussed, and consensus was reached through meetings held by the research team (A.-S. D, M.A., L.C., F.T.). An extraction template was created within Covidence, including the following fields: author, year, country, study design, study objective, population characteristics (age, gender, and diagnosis), intervention details, and primary outcomes. A dual extraction was conducted in parallel by two reviewers (A.-S.D. and F.T.), with the two versions consolidated by a third member (M.A).

## 3 Results

### 3.1 Literature search

The literature search identified 2,114 studies, which were imported for screening. One duplicate was detected and removed by Covidence. Following the title and abstract screening, 2,048 articles were deemed irrelevant and subsequently excluded. A total of 65 full-text studies were assessed for eligibility, of which 56 were excluded for the following reasons:

- **32** studies did not specifically focus on individuals exhibiting traits of BPD or related symptoms identified either through standardized self-report questionnaires or clinical assessment.
- **23** studies did not examine peer support interventions.
- **1** study was excluded as it was a thesis by articles, with its constituent papers already included in the current review.

**Figure 1:**
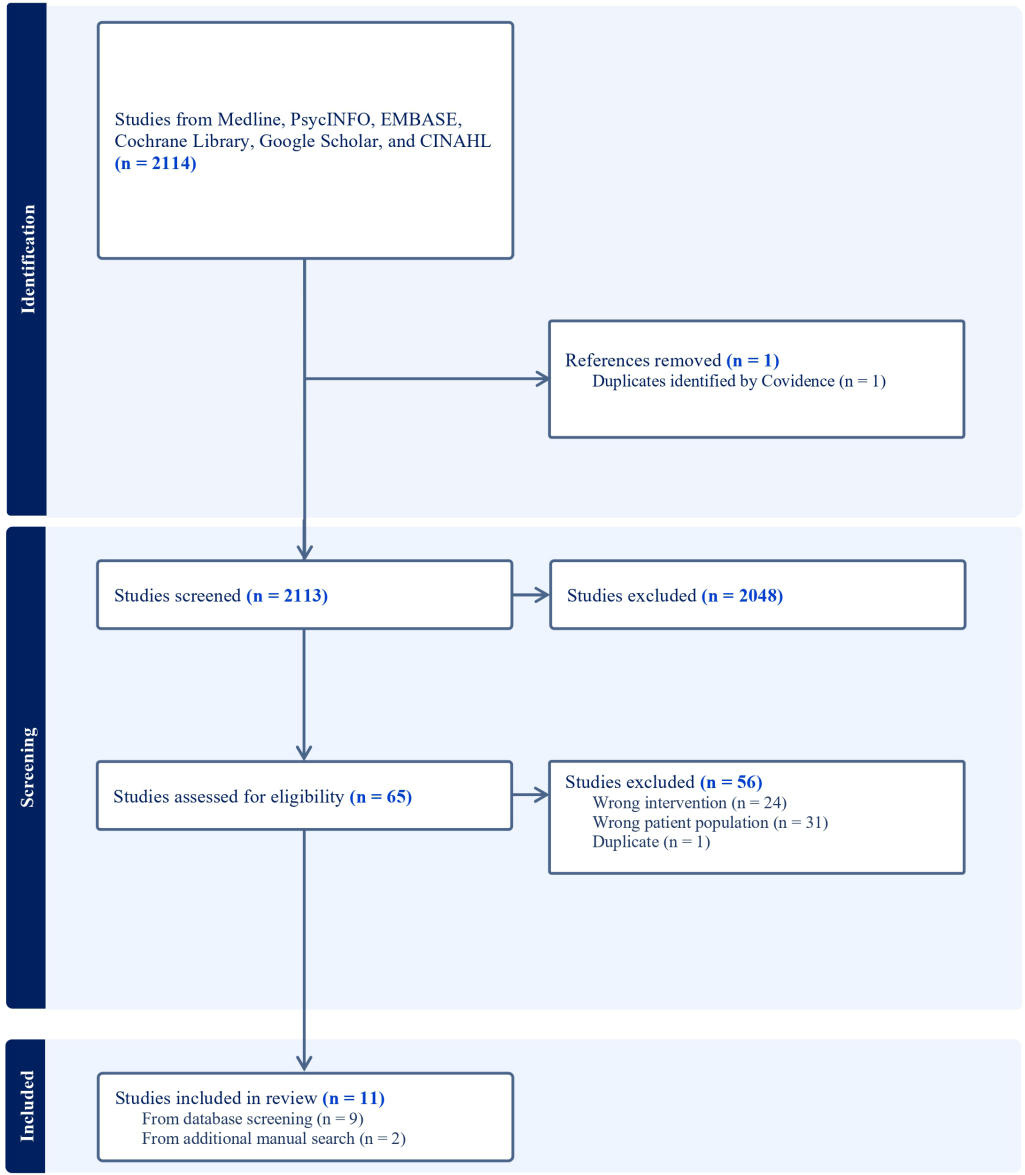
PRISMA-ScR flow diagram.

The detailed study selection process is illustrated in the PRISMA-ScR flow diagram (**Figure 1**), which was automatically generated by Covidence to provide a systematic overview of the inclusion and exclusion criteria applied throughout the screening process. Two relevant articles (35, 36) were identified through a final literature monitoring process conducted immediately prior to submission. As it met all inclusion criteria, it was added to the final set of included studies, resulting in a total of 11 articles (35–45).

### 3.2 Descriptive characteristics of included studies

The 11 studies included in this scoping review were published between 2019 and 2025. Detailed characteristics of each study are presented in **Supplementary File 2**. Australia was the most represented country (n = 5), followed by the United Kingdom (n = 2), with one study each from Mexico, South Africa, Spain, and France. A variety of methodological approaches were employed, most were qualitative studies (n = 5), using interviews and thematic analysis to explore participants’ experiences. One study adopted a mixed-methods design, combining both quantitative and qualitative analyses to provide a more comprehensive understanding of the phenomenon. Another study was a theoretical review (n = 1), synthesizing existing literature to develop conceptual frameworks or theoretical perspectives. One study was a descriptive report (n = 1) on the functioning of a peer support group, offering insights into its structure, processes, and impact. One naturalistic pre-post quantitative study (n = 1) evaluated the feasibility and acceptability of integrating a peer-support worker into an evidence-based group psychoeducation program. A feasibility randomized controlled trial protocol (n = 1) assessed the feasibility and acceptability of an intervention before conducting a larger-scale trial. Finally, one two-arm parallel-group randomized controlled trial compared a peer-clinician co-led intervention with treatment as usual.

### 3.3 Key findings

#### 3.3.1 Overview of peer support intervention modalities and characteristics

The peer support interventions included in this review varied considerably in their structure, delivery format, and therapeutic focus. Broadly, they can be categorized intro two main modalities based on their core features: structured and manualized peer-led programs, and community-based peer groups that range from semi-structured to unstructured formats. Five studies implemented structured and manualized peer-led programs (35–37, 42, 44). These interventions were delivered in group settings and followed a predefined curriculum or manual. Common components included psychoeducation, DBT-informed skills training, grounding or mindfulness exercises, and structured group discussions designed to foster emotion regulation and interpersonal functioning. Some programs also incorporated creative activities or parenting-specific modules tailored to the needs of particular subgroups. Among the structured, manualized peer-led programs, only three studies reported details on the selection, training, and supervision of peer-support workers (35, 36, 44). Across these programs, peer facilitators all had lived experience of borderline personality disorder and formal preparation for their roles. For example, facilitators had completed specialized university diplomas or certifications in mental-health peer work and Intentional Peer Support, and some held advanced training in approaches such as Good Psychiatric Management, Dialectical Behavior Therapy, counselling, narrative therapy, or art psychotherapy. Several also possessed professional credentials (e.g., registered art psychotherapist) and extensive advocacy experience. The remaining studies (n = 6) (38–41, 43, 45) described community-based or semi-structured peer groups. These interventions were more flexible in design, emphasizing mutual support, emotional validation, and the sharing of lived experience within a safe and non-judgmental environment. Unlike the more structured programs, they were less focused on skill acquisition and were more oriented toward fostering relational connection, normalization emotional distress, and reducing stigma.

The delivery settings of the peer support interventions varied across studies. Most interventions were conducted in group formats, with six studies (n = 6) delivered online either fully virtual (via videoconferencing platforms or online forums) or in hybrid formats combining in-person and remote participation (36, 38, 40, 42, 44, 45). These approaches were particularly common in studies conducted during or after the COVID-19 pandemic. Others were delivered in-person, either in clinical (n = 4) (35, 37, 39, 41) or community-based environments (n = 1) (43).

There was also substantial variability in the structures and scheduling of the peer support interventions, reflecting both the diversity of therapeutic models and some contextual constraints. Weekly sessions were the most reported format (n = 7), with sessions typically lasting 2 hours (35–38, 41, 42, 44). Among these, two programs were delivered over 10 weeks (n = 2), one extended to 18 weeks (n = 1), and another was designed as a 6-week program with 2-hour meetings sessions (n = 1). One study reported 90-minute sessions over a 6-week period (n = 1). Some interventions employed bi-weekly sessions or offered flexible frequencies depending on service structure or group dynamics (n = 2). In addition, three interventions adopted a continuous or open-ended format, particularly in virtual settings, allowing participants to access support on an ongoing basis without predefined timelines (40, 43, 45). These included asynchronous or user-initiated platforms with variable frequency and session lengths based on individual participation. For one study (39), details regarding frequency or duration of sessions were not fully specified, limiting direct comparison.

The content of the peer support interventions reviewed was diverse, reflecting the multifaceted needs of individuals with BPD. Psychological education was the most frequently integrated component (n = 8), aiming to enhance participants’ understanding of BPD and promote functioning (35–39, 42–44). Although DBT principles were explicitly referenced in only one study, several interventions included skill-building activities (n = 8), including training in emotion regulation, communication strategies, and mindfulness-based exercises to strengthen self-regulatory capacities (35–39, 41–43). Emotional support and validation (n = 9), often facilitated through the sharing of lived experience, were also central components, contributing to a sense of interpersonal safety and normalization of distress (35, 36, 38–43, 45).

#### 3.3.2 Peer support intervention outcomes

Among BPD individuals, reductions in BPD-related symptoms were reported in all three studies providing quantitative data (35, 36, 44). In a pre–post design without a control group (35), BPD symptom scores decreased significantly from baseline to post-intervention, and this reduction was maintained at the one-month follow-up. Total disability scores also declined significantly, although this effect was not sustained at follow-up, while social functioning improved and remained stable. In a randomized controlled trial (36), participants in the AIR Peers group demonstrated significant reductions in BPD symptom severity and improvements in general mental health compared to the control group. The proportion of participants meeting diagnostic criteria for BPD also declined markedly from baseline to follow-up, and participants reported high satisfaction with the program. Finally, another pre–post study without a control group (44) found significant improvements in emotion-regulation skills across all subscales following the intervention. In studies reporting qualitative data, consumers of peer support reported improvements in coping skills (n = 4), including the development of healthier emotional regulation strategies and enhanced self-reflective capacities (38, 41, 42, 44). Engagement in peer support was also associated with reduced feelings of isolation and decreased perceptions of stigma (n = 3) (38, 42, 44) Participants also reported feeling understood and validated in their lived experiences (n = 5), emphasizing the role of peer connection in creating a sense of acceptance and emotional safety (38–40, 42, 44). Peer support was further associated with increased hope (n = 3) (38, 39, 44), as well as enhanced empowerment and self-confidence (n = 5) (38, 39, 41, 42, 44), contributing to individuals feeling more in control of their recovery trajectories and capable of change. Despite limited quantitative evidence of symptom improvement, peer support appears to facilitate core aspects of subjective recovery, including improved interpersonal functioning, emotional coping, and perceived self-efficacy.

In two studies, outcomes for peer workers were also reported (38, 39). Peer workers described experiencing meaningful benefits, including personal growth (n = 2), as they developed new insights and evolved through their roles. In both studies, many reported an increased sense of purpose and self-worth derived from supporting others. These findings underscore the mutual benefits of peer support interventions, emphasizing their potential to improve emotional regulation, social connectedness, and personal empowerment for both consumers and peer facilitators in BPD.

#### 3.3.3 Challenges identified

While peer support interventions for individuals with BPD offer meaningful psychosocial benefits, several challenges that may impact their effectiveness and sustainability were documented. Emotional vulnerability and burnout were the most frequently reported issues (n = 6), with both peer workers and consumers experiencing emotional overload or distress due to repeated exposure to others’ struggles (39–42, 44, 45). These findings highlight the emotional toll of peer support roles and the need for structured self-care strategies. Role clarity and boundary issues were also identified (n = 3) as some peer workers reported difficulty distinguishing between their personal recovery experiences and their responsibilities within the peer support setting, leading to role ambiguity and blurred relational boundaries (39, 41, 42). Online and digital challenges (n = 4) were noted in interventions delivered virtually, where participants reported difficulties maintaining engagement and emotional connection in remote formats (40, 42, 44, 45). Additionally, two studies emphasized the need for supervision and training, particularly in preparing peer workers to handle crises, moderate complex group dynamics, and manage their dual identities as helpers and individuals with lived experience (39, 41). The development of conflict resolution strategies was also identified as essential, given the occasional interpersonal tensions that may arise in peer support settings (41). One study raised concerns regarding misinformation or unhelpful advice circulating in unmoderated settings, underscoring the importance of structured guidance and evidence-based training (45). Reports of stigma from mental health professionals were also noted among some peer workers, underscoring the need for greater systemic acceptance of lived experience roles (39). Finally, some participants expressed discomfort or skepticism toward peer roles (n = 1), reflecting potential barriers to acceptance and trust in peer-facilitated interventions (36).

## 4 Discussion

This scoping review aimed to evaluate the potential benefits and challenges of peer support for individuals with BPD, and to examine the structural, logistical, and ethical dimensions of implementing such interventions. Overall, findings from the 11 included studies highlighted both the promise and complexity of peer-led approaches in the context of BPD. Peer support has previously been shown to be both feasible and acceptable for individuals with BPD (46). While only a few studies demonstrated quantitative reductions in BPD symptom severity, qualitative findings consistently pointed to subjective improvements in emotional regulation, social connectedness, and psychological empowerment, core domains affected in BPD. Reported outcomes among participants included enhanced coping abilities, reduced feelings of isolation, and increased hope, while peer workers described experiences of personal growth and a strengthened sense of purpose. However, only three studies reported reductions in symptom severity using quantitative measures, and just one employed a randomized controlled design, the only design that allows firm conclusions about effectiveness. The other two used pre–post pre-experimental designs, making it difficult to attribute observed improvements to the intervention, as alternative explanations remain plausible. The remaining studies primarily employed qualitative methodologies, which are crucial for capturing participants’ lived experiences that may be overlooked by standardized measures (47). However, integrating quantitative studies could contribute to a more comprehensive assessment of clinical change. As a result, the most consistently reported benefits of peer support pertain to subjective and relational dimensions of recovery, such as enhanced emotional connection, validation, and hope. These findings, although encouraging, should be interpreted with caution due to the predominance of qualitative designs and the methodological heterogeneity across studies, which complicates the generalizability and comparability of outcomes.

Our research highlights a growing interest in the implementation of peer support interventions for individuals with BPD, with empirical studies published between 2019 and 2025 across diverse geographic and clinical settings. Despite their promising potential, it remains crucial to consider possible challenges, such as the emotional vulnerability associated with supporting others, difficulties maintaining connections in virtual formats, and ambiguity regarding roles and responsibilities. Providing support can be emotionally demanding for peer support workers, potentially leading to burnout or exacerbation of their own mental health issues (48). Close interactions within peer support settings may also facilitate the spread of maladaptive behaviors, such as self-harm or substance use, particularly among adolescents (49). Moreover, peer support relationships can blur professional and personal boundaries, leading to role confusion and unhealthy dependencies (50). The lack of clinical oversight increases the risk of misinformation or inadequate responses to complex psychiatric symptoms. Conflicts between peers and the potential reinforcement of stigma are additional concerns. To address these concerns, standardized, manualized programs offer a clearer therapeutic framework. A more formal model, such as the *Peer Support for People with Personality Disorder: A 6-Session Peer- and Clinician-Co-Facilitated Group Program* (51) as implemented by Grenyer and al. (2025), illustrates how a manualized approach grounded in dialectical behavior therapy principles can provide a clear therapeutic framework and detailed session plans. Similarly, Blay et al. (2025) implemented Ridolfi’s program (52), grounded in Good Psychiatric Management (GPM), an evidence-based generalist treatment for BPD. Such standardizations not only help maintain focus on skill development and mutual support while minimizing drift into non-therapeutic or potentially harmful interactions but also facilitate comparisons across studies and strengthen the ability to draw robust conclusions about the effectiveness of peer-support programs for individuals with BPD. In-person delivery also appears preferable, as several studies have noted that virtual groups limit disclosure and hinder the development of trust. The presence of a trained mental-health professional serving as co-facilitator can further ensure that boundaries are respected and that appropriate intervention is available if participants experience acute distress. In addition, systematic support for peer workers is essential to prevent emotional overload; scheduled individual supervision or debriefing sessions between group meetings can help peers process participants’ narratives and reduce the risk of emotional burnout. Finally, targeted training is critical. A recent international Delphi study on core competencies for mental-health peer-support workers (53) identified five essential domains for initial training: (1) using lived experience as a professional asset to foster hope and empathy while maintaining clear boundaries; (2) ethical competence, including confidentiality, informed consent, and respect for autonomy; (3) promotion of peer-worker well-being through self-care and early recognition of burnout; (4) sustaining a recovery-focused rather than clinical role; and (5) strong communication skills such as active listening, conflict management, and collaborative problem solving.

Defining these domains is a first step. Their relevance becomes clearer when considered in relation to the BPD population, where emotional dysregulation and challenges in social connectedness are well-documented (5, 25). Social connectedness includes structural (i.e., the number, diversity, or frequency of social relationships), functional (i.e., the actual or perceived resources provided by relationships), and quality (i.e., the positive and negative aspects of social relationships) dimensions. While BPD symptoms are linked to decreased social connectedness, the opposing relationship has not been firmly established (25). Specialized psychotherapies effectively address emotional dysregulation and elements of interpersonal functioning, yet peer support may act as a complementary intervention in this area. However, there is currently no comparison between peer support and specialized psychotherapy for BPD. For depression, at least one meta-analysis indicates that peer support can produce effects comparable to psychotherapy (54), raising the question of its specific efficacy in BPD, a disorder fundamentally rooted in relational challenges. Furthermore, the potential combined effect of integrating peer support with psychotherapy remains unexplored, underscoring the need for further research to determine whether peer support can enhance the therapeutic impact of specialized interventions.

### 4.1 Literature gaps and limitations

Several methodological limitations must be considered when interpreting the findings. One key issue is the predominance of qualitative studies. While qualitative research offers rich, in-depth insight into participants’ experiences, peer-support establishment as an intervention based on evidence would benefit from complementary quantitative studies. Such studies could contribute to measuring clinical outcomes and comprehensively assessing the impact of these interventions. Additionally, most studies included lack robust methodological controls, such as randomized controlled trials or comparator groups. Without appropriate control conditions, it is challenging to distinguish between the specific effects of peer support and other external influences. Moreover, there is no standardized peer support intervention across studies, leading to significant variability in implementation. Differences in training, structure, and delivery methods complicate the comparison of results and their generalization. Only three studies provided detailed information on the selection, training, and supervision of peer-support workers; greater reporting of these elements would not only clarify best practices but also facilitate the development of standardized interventions. Another critical limitation of the included studies is the substantial variability in follow-up durations, ranging from short-term assessments to periods that are insufficient to capture sustained effects. This heterogeneity hinders the ability to draw firm conclusions regarding the long-term efficacy and sustainability of peer support interventions. Furthermore, these interventions are implemented within their specific cultural and healthcare system contexts. Factors such as access to specialized psychotherapy, the structure and availability of mental health services, and differences in healthcare infrastructure, shape both the implementation and its potential effectiveness of peer support (50). Additionally, technological factors, such as internet accessibility and digital literacy, influence the feasibility and engagement in online peer support programs, contributing further to variability in outcomes.

### 4.2 Study limitations and strengths

This scoping review presents several limitations that should be considered when interpreting its findings. First, the study is subject to selection and publication bias, as it relies on existing literature, which may disproportionately include studies with positive or significant results while excluding unpublished or inconclusive research (55, 56). Additionally, despite a comprehensive search strategy, relevant studies may have been overlooked due to language restrictions or limitations in database coverage. Another significant limitation is the limited empirical investigation into the mechanisms and effectiveness of peer support interventions for individuals with BPD. While several studies suggest potential benefits, few have systematically examined how and why these interventions might lead to positive outcomes. A notable strength of this review is the inclusion of grey literature, which enabled the identification of alternative forms of peer support, such as online forums. This broadened perspective highlighted that, beyond structured interventions led by professionals, individuals with BPD naturally tend to seek and form peer-based connections. The broad scope of the review also revealed a growing interest in peer support not only within academic research but also in community-based settings, underscoring the need for further empirical investigation. Moreover, the use of PRISMA-ScR guidelines and the JBI methodology for scoping reviews ensured methodological rigor and transparency throughout the selection, data extraction, and synthesis processes.

## 5 Conclusion

This scoping review provides the first comprehensive synthesis of peer support interventions for individuals with BPD, highlighting both their significant promise and the complexities inherent in their implementation. Across diverse modalities and settings, peer support was found to have promising benefits for people with BPD, with qualitative evidence consistently pointing to improvements in emotional regulation, social connectedness, and empowerment, domains central to recovery in BPD. Both participants and peer workers reported meaningful benefits, including enhanced coping skills, reduced isolation, increased hope, and personal growth.

However, the evidence base remains limited by methodological heterogeneity and a predominance of qualitative studies, with few rigorous quantitative evaluations of clinical outcomes. This constrains the generalizability of findings and underscores the need for future research employing robust, mixed methods designs to more precisely assess the clinical impact of peer support in this population. Despite increasing recognition of the value of lived experience and peer involvement in mental healthcare over recent decades, there remains a notable scarcity of systematic research into the mechanisms, effectiveness, and best practices of peer support for people with BPD.

Notably, several challenges were identified that may affect the sustainability and effectiveness of peer-led interventions. Emotional vulnerability, risk of burnout, role ambiguity, and difficulties maintaining engagement—especially in virtual settings—were recurrent themes. These findings underscore the necessity of structured supervision, clear role definitions, and comprehensive training for peer workers to ensure the safety and efficacy of these interventions. Moreover, the occasional presence of stigma from professionals and skepticism among participants points to the need for broader systemic acceptance and integration of peer roles within mental health services.

Nevertheless, this review provides an initial integration of current knowledge, highlighting both the promise and the challenges of peer-led interventions for individuals with BPD. It thus offers a foundation for future clinical and empirical studies aimed at clarifying the unique contributions, optimal implementation strategies, and long-term effects of peer support in this population.

## Data Availability

The original contributions presented in the study are included in the article/**Supplementary Material**. Further inquiries can be directed to the corresponding author.
