# Supplementary material for: Is peer support beneficial for individuals with borderline personality disorder? Exploring its potential and challenges – a scoping review
Source: Front Psychiatry. 2025 Oct 23;16:1663685. doi: 10.3389/fpsyt.2025.1663685 (PMC12590239; doi:10.3389/fpsyt.2025.1663685)
Supplement: Supplementary file 1 [file Table1.docx]

**Supplementary file 1**

*Summary of studies investigating peer support for adults living with borderline personality disorder*

| **Authors** | **Country** | **Study type** | **Objective(s)** | **Participants characteristics** | **Intervention characteristics** | **Main finding** |
| --- | --- | --- | --- | --- | --- | --- |
| (Barr et al. ,2022) | Australia | Qualitative | Determine the benefits, challenges and unique contributions regarding the peer worker role for consumers, peer workers and clinicians. | **Peer workers**   - n=5 - 100%F - M_age_=33±4.4   **Consumers with BPD**   - n=14 - 100%F - M_age_=36.2±9.2   **Clinicians**   - n=4 - 75%F - M_age_=36.9±8.7)   **Total:** n=23 | **Type:**  Peer support provided in outpatient mental health and independent peer-run services Combination of group support and individual meetings Virtual sessions **Frequency of Meetings:**  Weekly (92.9%) and bi-weekly (7.1%) programs lasting 10 weeks **Content:**  Education about BPD Shared experiences Skill sharing Art-based activities Social activities Resource information | **Consumer outcomes:**   - Feeling Understood - Increased Hope - Development of coping strategies skills - Reduced isolation - Authentic relationships - Empowerment   **Peer worker outcomes**:   - Personal Growth and Recovery - Sense of Purpose and Value   **Challenges:**  NAN |
| (Dahlenburg et al., 2024) | Australia | Qualitative | To explore participant experiences following engagement in a peer group program for individuals diagnosed with borderline personality disorder | **Consumers with BPD**   - n=22 - 87% cisgender F - 3% cisgender M - M_age_=42.6 ±11.9   **Total:** n=22 | **Type:**  Manualized peer group program  Co-facilitated by a person with lived experience of BPD and a senior mental health clinician Face-to-face and online sessions **Frequency of Meetings**:  Weekly sessions of 2 hours, 10 weeks  **Content:** Psychoeducation Creative activities  Sharing personal experiences Mindfulness and grounding exercises Skills development and emotional regulation strategies | **Consumer outcomes:**   - Increased self-confidence, empowerment, and improved coping strategies. - Connection with others - Feeling Understood - Reduction of Stigma - Creation of Safe Space   **Peer worker outcomes**: NAN  **Challenges:**   - Difficult to maintain a sense of connection in virtual formats - Emotional Vulnerability - Need for Clear Boundaries |
| (Jewell et al., 2022) | Australia | Qualitative + Quantitative measures | To evaluate the acceptability and potential effectiveness of a peer-led, art-based online skills program for individuals with borderline personality disorder and emotion dysregulation | **Consumers with BPD**   - n=31 - 89.5% identified with she/her pronouns - 10.5% identified with they/them - M_age_=33.6 ±9.6   **Total:** n=31 | **Type:**  Structured, manualized art-based skills program delivered online, facilitated by a peer mental health professional with lived experience of BPD  **Frequency of Meetings**:  18 weeks, 2 hours  **Content:**  Artmaking activities Psychoeducation based on dialectical behavior therapy (DBT) principles Self-expression and creative exercises Mindfulness and grounding activities | **Consumer outcomes:**   - Significant reduction in difficulties with emotion regulation with a large effect size (Cohen’s *d* = 1.77) - Increased Coping Skills - Enhanced Self-Understanding - Reducing feelings of isolation - Higher Self-Compassion - Hope for Recovery   **Peer worker outcomes**: NAN  **Challenges**:   - Barriers to Online Engagement - Emotional Vulnerability |
| (Baker et al., 2023) | United Kingdom | Randomized controlled trial protocol | To assess the feasibility, acceptability, and potential effectiveness of the BAP-EFL peer-led intervention for parents/caregivers with significant emotional and interpersonal difficulties | **Parents/caregivers with significant emotional and interpersonal difficulties**   - Age: Parents aged 18–65 years; | **Type:**  Being a Parent–Enjoying Family Life (BAP-EFL): peer-led, manualized parenting program focusing on emotion regulation, positive parenting, and reflective functioning Group-based peer support facilitated by trained peer group leaders **Frequency of Meetings**:  10-week  **Content:**  Parenting psychoeducation Emotion regulation strategies Positive parenting techniques Goal setting and self-care exercises Reflective discussions and peer support activities | NAN |
| (Barr et al. ,2020) | Australia | Qualitative | To explore perceptions and models of peer support for individuals with borderline personality disorder (BPD) from the perspectives of consumers, carers, and mental health professionals | **Peer workers**   - n=12 - 100% F   **Consumers with BPD**   - n=12 - 66.7% F   **Clinicians**   - n=12 - 58.3% F   **Total:** n=36 | **Type:**  Peer support provided through structured and informal models Included consumer peer workers and carer peer workers supporting individuals with BPD and their carers Combination of individual and group peer support  **Frequency of Meetings**:  NAN  **Content:**  Emotional support and validation Sharing lived experiences Psychoeducation about BPD Skills coaching and support with therapy-related tasks | **Consumer outcomes**:   - Hope and Connection - Validation and Understanding - Empowerment   **Peer worker outcomes**:   - Personal Growth - Professional Development   **Challenges:**   - Role Clarity: Ambiguity regarding the roles of consumer peer workers - Peer workers experienced stigma from mental health professionals - Emotional Burden for peer worker |
| (Bond et al., 2019) | United Kingdom | Qualitative | Explore how members and facilitators of a borderline personality disorder (BPD) self-help group interact and make sense of their experiences to identify what aspects of these interactions are helpful | **Members of a BPD self-help group and the co-facilitator**   - 100% F - n=10 | **Type:**  A peer-led self-help group for individuals with BPD, focusing on emotional support, sharing experiences, and mutual aid  **Frequency of Meetings**:  Weekly 2-hour, for 9 weeks  **Content:**  Emotional sharing and validation Experiential knowledge exchange Peer support discussions Reflection activities and coping strategies | **Consumer outcomes:**   - Sense of Belonging - Empowerment - Healthier coping mechanisms for managing intense emotions - Improved Self-Reflection   **Peer worker outcomes**:  NAN  **Challenges:**   - Emotional Vulnerability - Occasional interpersonal tensions - Need for clear boundaries and conflict resolution strategies within the group |
| (Islas León, 2022) | Mexico | Narrative review | To explore the feasibility of virtual self-help groups for individuals with borderline personality disorder (BPD), particularly in response to the COVID-19 pandemic | NAN | **Type:**  Virtual self-help groups as a complementary intervention to traditional therapies for BPD Focus on the use of online platforms like WhatsApp, FONO HELP, and other virtual support tools **Frequency of Meetings**:  Continuous, as virtual platforms allow ongoing interactions **Content:**  Emotional support Sharing personal experiences Group discussions and mutual aid | **Consumer outcomes:**  NAN  **Peer worker outcomes**:  NAN  **Challenges:**   - Lack of Professional Moderation: risk of misinformation or unhelpful advice being shared among peers - Emotional Overload: experience emotional distress from exposure to others - Boundary Issues: The absence of face-to-face interactions may complicate |
| (Cordeiro, 2023) | South Africa | Descriptive report *(grey literature)* | To describe the establishment and benefits of a peer support group for young adults with borderline personality disorder (BPD), aiming to provide a non-judgmental space for emotional support and coping strategies | **Young adults diagnosed with BPD**   - Age: 18-35 years | **Type:**  A structured support group specifically for young adults diagnosed with BPD **Frequency of Meetings**:  Continuous support as long as the individual remains engaged **Content:**  Emotional validation and support Psychoeducation about BPD Development of communication and problem-solving skills | NAN |
| (Figueras Bates, 2023) | Spain | Qualitative | To compare how emotions are expressed, and empathy is displayed in two online mental health forums: one for borderline personality disorder (BPD) and one for eating disorders (ED) | **Anonymous Reddit users**  **Total:** 303 comments analyzed from the BPD forum | **Type:**  Online peer support through Reddit forums  Virtual/online peer support  **Frequency of Meetings**:  Asynchronous interactions, Ongoing, user-initiated discussions without a fixed schedule **Content:**  Sharing personal experiences Emotional validation Providing advice based on lived experiences Offering informational support and coping strategies | **Consumer outcomes**:   - Emotional Validation - Sense of Belonging   **Peer worker outcomes**:  NAN  **Challenges:**   - Emotional Overload - Boundary Issues: The absence of formal structure in online forums limited deeper personal connections compared to face-to-face peer support. |
| (Blay et al., 2025) | France | Naturalist study  Feasibility study (pre-post, uncontrol) | To assess the feasibility and acceptability of integrating a peer-support worker into an evidence-based BPD program | **Adults diagnosed with borderline personality disorder**   - n=46 - 76.09% M M_age_=32.1±9.4 | **Type:**  Group psychoeducation based on Good Psychiatric Management, co-led by a clinical psychologist  **Frequency of Meetings**: Weekly (6 weeks), 90 min per session **Content:** Psychoeducation, sharing of lived experience, skill training, listening | **Consumer outcomes:**   - Significant reduction in ZAN-BPD score and disability post-group - Increased satisfaction in social functioning   **Peer worker outcomes**:   - NAN   **Challenges**:   - NAN |
| (Grenyer et al., 2025) | Australia | RCT | Evaluate the effectiveness of a brief, co-facilitated peer and clinician-led group therapy program (AIR Peers) for individuals with borderline personality disorder (BPD). | **Adults with BPD**   - n=83 - 88%F - M_age_=36±11 | **Type:**  AIR Peers program: manualized group therapy co-led by a peer worker with lived BPD experience and a clinical psychologist. Virtual/online peer support groups **Frequency of Meetings**:  6-week of 2 hours session **Content:** Psychoeducation Shared lived experience Skills training Supportive listening Group discussion Recovery-oriented activities Emotional support | **Consumer outcomes:**   - Significant reduction in BPD symptom severity - Improved mental health (MHI-5 scores) - 86% rated the treatment as helpful - 97% would recommend the program   **Peer worker outcomes**:  NAN  **Challenges:**   - Consumers expressed discomfort or skepticism, sometimes perceiving the peer worker as less professional than the clinician |

**Supplementary file 2.**

The search strategy for each database and for the grey literature search

| **Peer support** | **Bordeline personality disorder** | **Symptoms** |
| --- | --- | --- |
| **Descripteurs (MeSH)**  "Peer Group"[Mesh]  "Peer Influence"[Mesh]  "Self-Help Groups"[Mesh:NoExp]  Peer(s)  AND  "Patient Education as Topic"[Mesh:NoExp]  "Patient Care Team"[Mesh:NoExp]  "Psychosocial Intervention"[Mesh]  **Mots clés (titres/résumés)**  Peer group(s)  Peer influcence  Self-help group(s)  Support group(s)  Peer support(s)  Peer caregiver(s)  Peer mentor(s)  Peer network(s)  Peer education  **Chercher avec l’adjacence*  Peer-led  Peer-mediated  Peer-assisted  Peer-based  Peer role model(s)  Peer specialist(s)  Peer facilitator(s)  Peer-delivered  Peer(s)  ADJ3  Intervention(s)  Care  Program(s)  Service(s) | **Descripteurs (MeSH)**  "Borderline Personality Disorder"[Mesh]  "Personality Disorders"[Mesh:NoExp]  **Mots clés (titres/résumés)**  Personality disorder(s)  BPD  Borderline | **Descripteurs (MeSH)**  "Anxiety, Separation"[Mesh]  "Identity Crisis"[Mesh]  "Impulsive Behavior"[Mesh:NoExp]  "Self-Control"[Mesh]  "Anger"[Mesh]  "Irritable Mood"[Mesh]  **Mots clés (titres/résumés)**  fear of abandonment  fear of separation  separation anxiety  abandonment anxiety  identity disturbance(s)  identity crisis  impulsivity  impulsiveness  impulsive  affective instability(ies)  mood instability(ies)  emotion(al) instability(ies) emotion(al) dysregulation  emotion(al) regulation  self-regulation(s)  self-control  mood reactivity  affective reactivity  anger management  managing anger  anger control(lling)  inappropriate anger  irritable mood  interpersonal sensitivity(ies)  interpersonal instability(ies) |

20 last years (2003-2024)

English and French

**Concept 1**

"Peer Group"[Mesh] OR "Peer Influence"[Mesh] OR "Self-Help Groups"[Mesh:NoExp] OR (peer*[TIAB] AND ("Patient Education as Topic"[Mesh:NoExp] OR "Patient Care Team"[Mesh:NoExp] OR "Psychosocial Intervention"[Mesh])) OR "Peer group"[TIAB:~3] OR "peer groups"[TIAB:~3] OR "Peer influcence"[TIAB:~3] OR "Self-help group"[TIAB:~3] OR "self-help groups"[TIAB:~3] OR "Support group"[TIAB:~3] OR "support groups"[TIAB:~3] OR "Peer support"[TIAB:~3] OR "peer suppports"[TIAB:~3] OR "Peer caregiver"[TIAB:~3] OR "peer caregivers"[TIAB:~3] OR "Peer mentor"[TIAB:~3] OR "peer mentors"[TIAB:~3] OR "Peer network"[TIAB:~3] OR "peer networks"[TIAB:~3] OR "Peer education"[TIAB:~3] OR "Peer-led"[TIAB] OR "Peer-mediated"[TIAB] OR "Peer-assisted"[TIAB] OR "Peer-based"[TIAB] OR "Peer role model*"[TIAB] OR "Peer specialist*"[TIAB] OR "Peer facilitator*"[TIAB] OR "Peer-delivered"[TIAB] OR "peer intervention"[TIAB:~3] OR "peer interventions"[TIAB:~3] OR "peers intervention"[TIAB:~3] OR "peers interventions"[TIAB:~3] OR "peer care"[TIAB:~3] OR "peers care"[TIAB:~3] OR "peer service"[TIAB:~3] OR "peer services"[TIAB:~3] OR "peers service"[TIAB:~3] OR "peers services"[TIAB:~3] OR "peer program"[TIAB:~3] OR "peer programs"[TIAB:~3] OR "peers program"[TIAB:~3] OR "peers programs"[TIAB:~3]

**Concept 2**

"Borderline Personality Disorder"[Mesh] OR "Personality Disorders"[Mesh:NoExp] OR "personality disorder*"[TIAB] OR BPD[TIAB] OR borderline[TIAB]

**Concept 3**

"Anxiety, Separation"[Mesh] OR "Identity Crisis"[Mesh] OR "Impulsive Behavior"[Mesh:NoExp] OR "Self-Control"[Mesh] OR "Anger"[Mesh] OR "Irritable Mood"[Mesh] OR "fear abandonment"[TIAB:~2] OR "fear separation"[TIAB:~2] OR "separation anxiety"[TIAB:~2] OR "abandonment anxiety"[TIAB:~2] OR "identity disturbance"[TIAB:~2] OR "identity disturbances"[TIAB:~2] OR "identity crisis"[TIAB:~2] OR impulsiv*[TIAB] OR "affective instabilit*"[TIAB] OR "mood instabilit*"[TIAB] OR "emotional instabilit*"[TIAB] OR "emotion instabilit*"[TIAB] OR "emotions instabilit*"[TIAB] OR "emotional dysregulation"[TIAB] OR "emotion dysregulation"[TIAB] OR "emotions dysregulation"[TIAB] OR "emotional regulation"[TIAB] OR "emotion regulation"[TIAB] OR "emotions regulation"[TIAB] OR self-regulation*[TIAB] OR self-control[TIAB] OR "mood reactivity"[TIAB] OR "affective reactivity"[TIAB] OR "anger management"[TIAB:~3] OR "managing anger"[TIAB:~3] OR "anger control"[TIAB:~3] OR "controlling anger"[TIAB:~3] OR "inappropriate anger"[TIAB] OR "irritable mood"[TIAB] OR "interpersonal sensitivit*"[TIAB] OR "interpersonal instabilit*"[TIAB]

**Unrestricted vocabulary**

(peer ADJ3 (group* OR influence OR support* OR caregiver* OR mentor* OR network* OR education)) OR (self-help ADJ3 group*) OR (support ADJ3 group*) OR peer-led OR peer-mediated OR peer-assisted OR peer-based OR "peer role model*" OR "peer specialist*" OR "peer facilitator*" OR "peer-delivered" OR (peer* ADJ3 (intervention* OR care OR program* OR service*))

"personality disorder*" or BPD or Borderline

((fear or anxiety) ADJ2 (abandonment OR separation)) OR (identity ADJ2 (disturbance* OR crisis)) OR impulsiv* OR "affective instabilit*" OR "mood instabilit*" OR "emotional instabilit*" OR "emotion* instabilit*" OR "emotion* dysregulation" OR "emotion* regulation" OR self-regulation* OR self-control OR "mood reactivity" OR "affective reactivity" OR (anger ADJ3 (management OR managing OR control OR controlling)) OR "inappropriate anger" OR "irritable mood" OR "interpersonal sensitivit*" OR "interpersonal instabilit*"
